# HNG, A Humanin Analogue, Promotes Hair Growth by Inhibiting Anagen-to-Catagen Transition

**DOI:** 10.3390/ijms21124553

**Published:** 2020-06-26

**Authors:** Sung Min Kim, Jung-Il Kang, Hoon-Seok Yoon, Youn Kyung Choi, Ji Soo Go, Sun Kyung Oh, Meejung Ahn, Jeongtae Kim, Young Sang Koh, Jin Won Hyun, Eun-Sook Yoo, Hee-Kyoung Kang

**Affiliations:** 1 Department of Medicine, School of Medicine, Jeju National University, 102 Jejudaehakno, Jeju 63243, Korea; seongmin85@naver.com (S.M.K.); jikang0024@jejunu.ac.kr (J.-I.K.); hsyoon717@jejunu.ac.kr (H.-S.Y.); choiyk@jejunu.ac.kr (Y.K.C.); raccunjis@daum.net (J.S.G.); seonkyeong@gmail.com(S.K.O.); yskoh7@jejunu.ac.kr (Y.S.K.); jinwonh@jejunu.ac.kr (J.W.H.); eunsyoo@jejunu.ac.kr (E.-S.Y.); 2 Department of Animal Science, College of Life Science, Sangji University, Wonju 26339, Korea; meeahn20@sangji.ac.kr; 3 Department of Anatomy, Kosin University College of Medicine, Busan 49267, Korea; kimjt78@kosin.ac.kr; 4 Jeju Research Center for Natural Medicine, Jeju National University, 102 Jejudaehakno, Jeju 63243, Korea

**Keywords:** humanin, hair growth, dermal papilla cells, anagen, VEGF

## Abstract

The hair follicle goes through repetitive cycles including anagen, catagen, and telogen. The interaction of dermal papilla cells (DPCs) and keratinocytes regulates the hair cycle and hair growth. Humanin was discovered in the surviving brain cells of patients with Alzheimer’s disease. HNG, a humanin analogue, activates cell growth, proliferation, and cell cycle progression, and it protects cells from apoptosis. This study was performed to investigate the promoting effect and action mechanisms of HNG on hair growth. HNG significantly increased DPC proliferation. HNG significantly increased hair shaft elongation in vibrissa hair follicle organ culture. In vivo experiment showed that HNG prolonged anagen duration and inhibited hair follicle cell apoptosis, indicating that HNG inhibited the transition from the anagen to catagen phase mice. Furthermore, HNG activated extracellular signal-regulated kinase (Erk)1/2, Akt, and signal transducer and activator of transcription (Stat3) within minutes and up-regulated vascular endothelial growth factor (VEGF) levels on DPCs. This means that HNG could induce the anagen phase longer by up-regulating VEGF, which is a Stat3 target gene and one of the anagen maintenance factors. HNG stimulated the anagen phase longer with VEGF up-regulation, and it prevented apoptosis by activating Erk1/2, Akt, and Stat3 signaling.

## 1. Introduction

The hair found in mammalian skin has important functions like physical protection, body temperature maintenance, and sensory perception. It also has a cosmetic effect in humans. Recently, there have been increasing numbers of people suffering from hair loss, also called alopecia. Nevertheless, only two drugs have been approved for hair loss treatment by the FDA: finasteride and minoxidil. However, finasteride and minoxidil use has limited utility for curing alopecia due to its transient action and adverse effects [1]. Therefore, there is a high demand to develop new remedies for hair loss.

The hair follicle that resides in all layers of the skin is a dynamic mini-organ. The hair follicle is made up of 20 cell types, and it goes through repetitive cycles that interact with each other: growth (anagen), apoptosis-driven regression (catagen), and quiescence (telogen) [2,3]. If the transition of telogen to anagen is faster or transition of anagen to catagen is slower, miniaturization of hair and hair loss would be reduced [4]. Among cells in the hair follicle, dermal papilla cells (DPCs), a mesenchymal population located in the base of hair follicle, play an important role in follicle morphogenesis, hair shaft diameter, and hair cycle regulation by epithelial-mesenchymal interaction [5,6,7,8,9]. In particular, the number of DPCs is important for maintaining the anagen phase, hair size, and hair shape [3,10,11,12]. Transitioning from telogen-anagen, the dermal papilla is recovered with the same number of cells in previous anagen by cell division within the dermal papilla itself and influx of dermal sheath cells to dermal papilla [4]. Most alopecia cases are characterized by a destructive hair cycle due to a failure to maintain DPC number by the increase in efflux from the dermal papilla and/or the reduction of influx from the dermal sheath [3,13]. Therefore, maintaining DPC number, increasing DPC proliferation, or both is necessary to cure alopecia.

Humanin is a polypeptide consisting of 24 amino-acids [14,15,16,17]. It was first discovered in the surviving brain cells of patients who have Alzheimer’s disease. It has reported that humanin protects cells from apoptosis in the brain of the patients [14,18]. HNG is an analogue of humanin with the 14th-serine substituted for a glycine, and it is 1000 times more potent than humanin [14,18,19,20]. It has been reported that 57 proteins are activated by HNG treatment. These proteins activated by HNG are categorized into different molecular functional types: kinase, transcription regulator, translational regulator, transmembrane receptor, transporter, and so on. In addition, these proteins are significantly correlated with cell growth, proliferation, and the cell cycle [21]. HNG has neuroprotective effects by activating extracellular signal-regulated kinase (ERK)1/2, Akt, and signal transducer and activator of transcription 3 (STAT3) signaling in neuroblastoma cells [21]. On the other hand, minoxidil induces DPC proliferation by activating ERK1/2 and Akt [22].

However, the effect of HNG on the regulation of hair growth has not been studied. Here, we demonstrated the promoting effect and the action mechanisms of HNG on hair growth. We found that HNG up-regulated vascular endothelial growth factor (VEGF), a potent stimulator of anagen phase, by activating Erk1/2, Akt, and Stat3, which seems to stimulate hair growth. HNG increased the proliferation of DPCs in vitro and the elongation of the hair shaft ex vivo, and it stimulated the duration of the anagen phase in vivo, which suggests that HNG would improve various types of alopecia.

## 2. Results

### 2.1. HNG Increases the Proliferation of Dermal Papilla Cells

**Figure 1:**
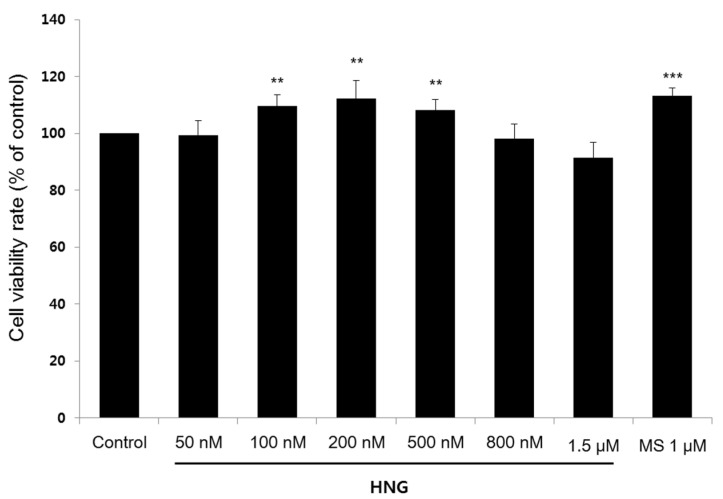
HNG increases the proliferation of dermal papilla cells (DPCs). Immortalized rat DPCs (0.5 × 10^4^ cells/mL) were seeded in 96-well plates. DPCs were treated with HNG 50 nM to 1.5 μM for 72 h. Cell proliferation was measured using the MTT assay. All experiments were performed in triplicate. Data are shown as the mean ± the S.D. ** *p* < 0.01, *** *p* < 0.001 vs. control.

We performed the proliferation assay using 3-[4,5-dimethylthiazol-2-yl]-2,5-diphenyltetrazolium bromide (MTT) solution to examine whether HNG increases the proliferation of DPCs. Treatment with HNG for 72 h significantly increased the proliferation of DPCs by 109.6 ± 3.9%, 112.2 ± 6.4%, and 108.1 ± 3.8% at 100 nM, 200 nM, and 500 nM, respectively (Figure 1). The proliferation of DPCs treated with 1 μM minoxidil sulfate (MS), an active metabolite of minoxidil as positive control, also increased significantly by 113.2 ± 2.9% as reported (Figure 1) [22]. HNG seems to stimulate hair growth via DPC proliferation, which is important in hair growth phase extension.

### 2.2. HNG Increases Hair Shaft Elongation Ex Vivo

**Figure 2:**
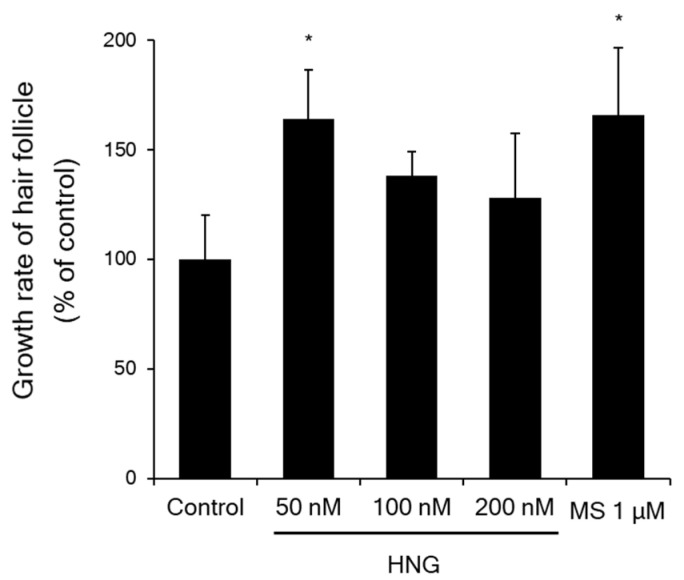
HNG increases hair shaft elongation. Vibrissa follicles in anagen phase were treated with HNG from 50 to 200 nM for 21 days. Media was refreshed every three days. The mean growth rate of the vibrissa follicle of control group on the 21st day was set as 100%. Data are shown as the mean ± S.E.M. * p < 0.05 vs. control.

**Figure A1:**
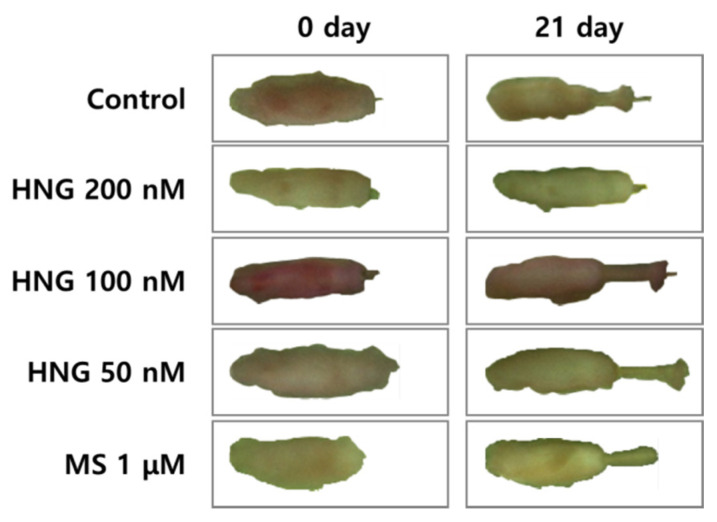
Photographs of the cultured rat vibrissa follicles acquired using a stereomicroscope. Rat vibrissa follicles were isolated and cultured with the indicated concentrations of HNG or MS for 21 days.

To investigate whether HNG promotes hair shaft elongation, vibrissa follicles in anagen phase were isolated and treated with or without HNG (50, 100, and 200 nM), and with MS 1 μM, respectively, in groups (*n* = 7). HNG 50 nM significantly increased hair shaft elongation by 163.9 ± 22.7% on the 21st day (Figure A1 and Figure 2). MS 1 μM was used as the positive control, which also significantly increased hair fiber elongation by 166.0 ± 30.7% on the 21st day as reported (Figure A1 and Figure 2) [22,23]. Vibrissa follicles treated with HNG or MS showed early catagen shape, while those of untreated controls showed late catagen shape on the 21st day (Figure A1) [24]. HNG promotes hair shaft elongation by delaying catagen progression ex vivo.

### 2.3. HNG Promotes In Vivo Hair Growth

**Figure 3:**
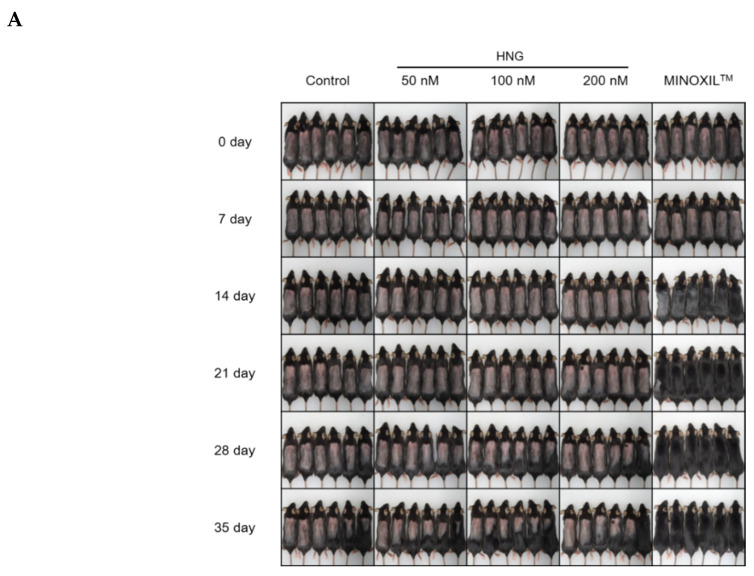

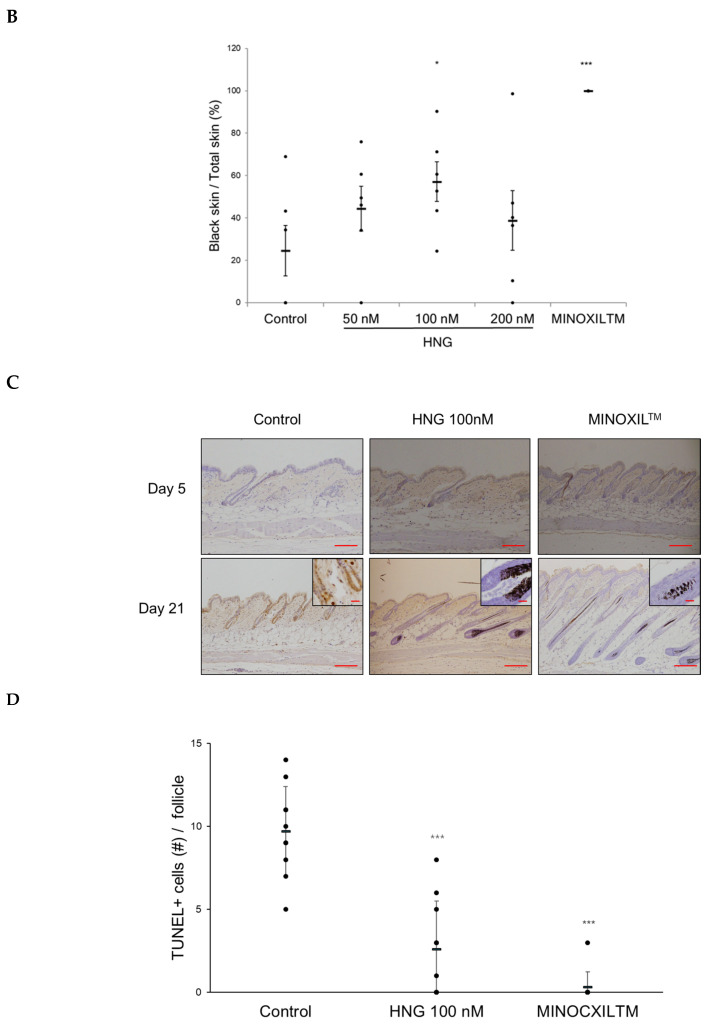

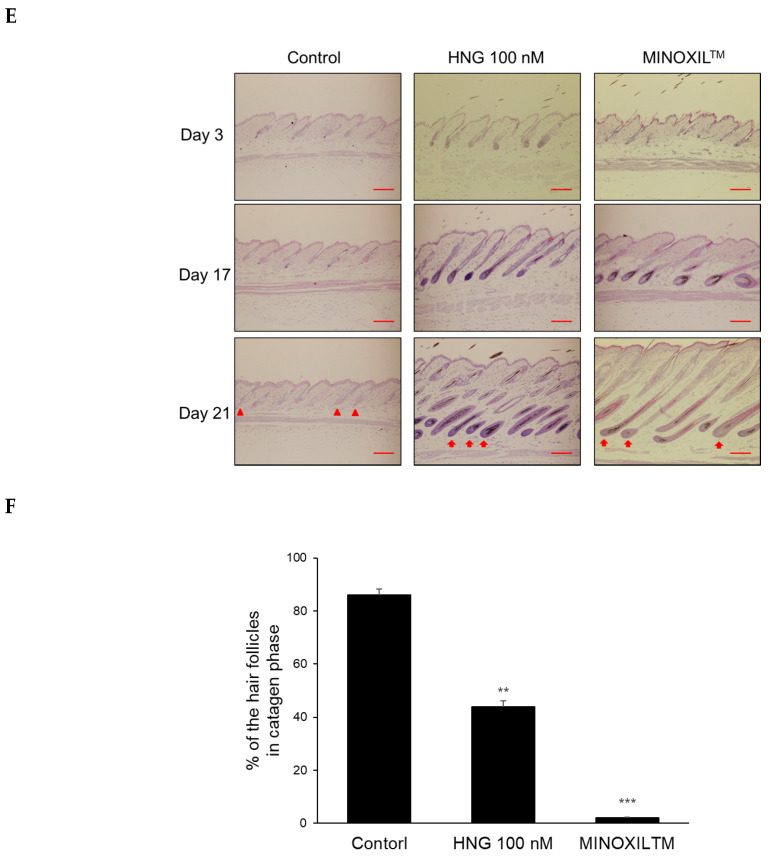
HNG stimulates the duration of the anagen phase in vivo model. After depilation, the dorsal skin of the mice was topically treated with vehicle, HNG, and MINOXIL™ once a day for 35 days. (**A**) The dorsal skin of C57BL/6 mice was photographed from day 0 to the 35th day on every 7th day after depilation. (**B**) Quantified hair growth in control, HNG 50, 100, 200 nM, and MINOXIL™ groups on the 35th day using dot matrix planimetry. (**C**) Colorimetric Tdt-mediated dUTP-Dig nick and labeling (TUNEL) assay performed. Scale bars represent 100 μm, but 200 μm in smaller figures. (**D**) Quantitative analysis of TUNEL-positive cells on the 21st day in (**C**). Mean values ± S.D. *** p < 0.001 vs. control. At least 10 hair follicles were examined. (**E**) Hematoxylin and eosin staining. Scale bars represent: 100 μm; anagen hair follicle indicated by arrow; catagen hair follicle indicated by arrowhead. (**F**) Quantification (%) of catagen hair follicle in (**E**) using histomorphometry. Hair follicles (*n* ≥ 100) on the 21st day were examined. Mean values ± S.D. ** *p* < 0.01 vs. control, *** *p* < 0.001 vs. control.

To examine whether HNG stimulates anagen phase in vivo, we shaved back skin of C57/BL6 on the 50th day after birth and treated topically on the dorsal back with vehicle as control (PBS and ethanol in 1:1 (*v*/*v*)), HNG (50, 100, and 200 nM), and the MINOXIL™ (minoxidil 5%) as positive control, respectively (Figure 3A). As expected, treatment with MINOXIL™ (minoxidil 5%) promoted hair growth and presented a statistically significant difference within 14 days (Figure 3A,B). Interestingly, treatment with HNG (100 nM) promoted hair growth and presented a statistically significant difference within 35 days (Figure 3A,B). However, there was no significant difference in the beginning of the anagen phase between the group treated with HNG and the control group treated with vehicle. It has been reported that catagen-associated changes like the skin color, thickness, and apoptosis of the cells in hair follicles are first seen on the 17th day when entering catagen after anagen phase [25,26]. As such, we investigated apoptosis of the cells in the hair follicle on the 21st day. Surprisingly, the number of the apoptotic cells in HNG 100 nM treatment was prominently less than in vehicle treatment as control (Figure 3C,D). HNG 100 nM and MINOXIL™ (minoxidil 5%) increased the thickness of the entire dermis and subcutis of the dorsal skin on the 17th and 21st days, which indicate that HNG stimulates anagen phase duration (Figure 3E). To access the impact of HNG on the hair-cycle-related morphology of hair follicles, we performed quantitative histomorphometry based on the shape and size of dermal papilla (Figure 3F) [25,27]. Catagen hair follicles significantly decreased by HNG or MINOXIL™ treatment (control, 86 ± 2.4%; HNG 100 nM, 44 ± 2.1%; MINOXIL™, 2.0 ± 0.2%; Figure 3F).

### 2.4. HNG Activates Erk1/2, Akt, and Stat3 in DPCs

**Figure 4:**
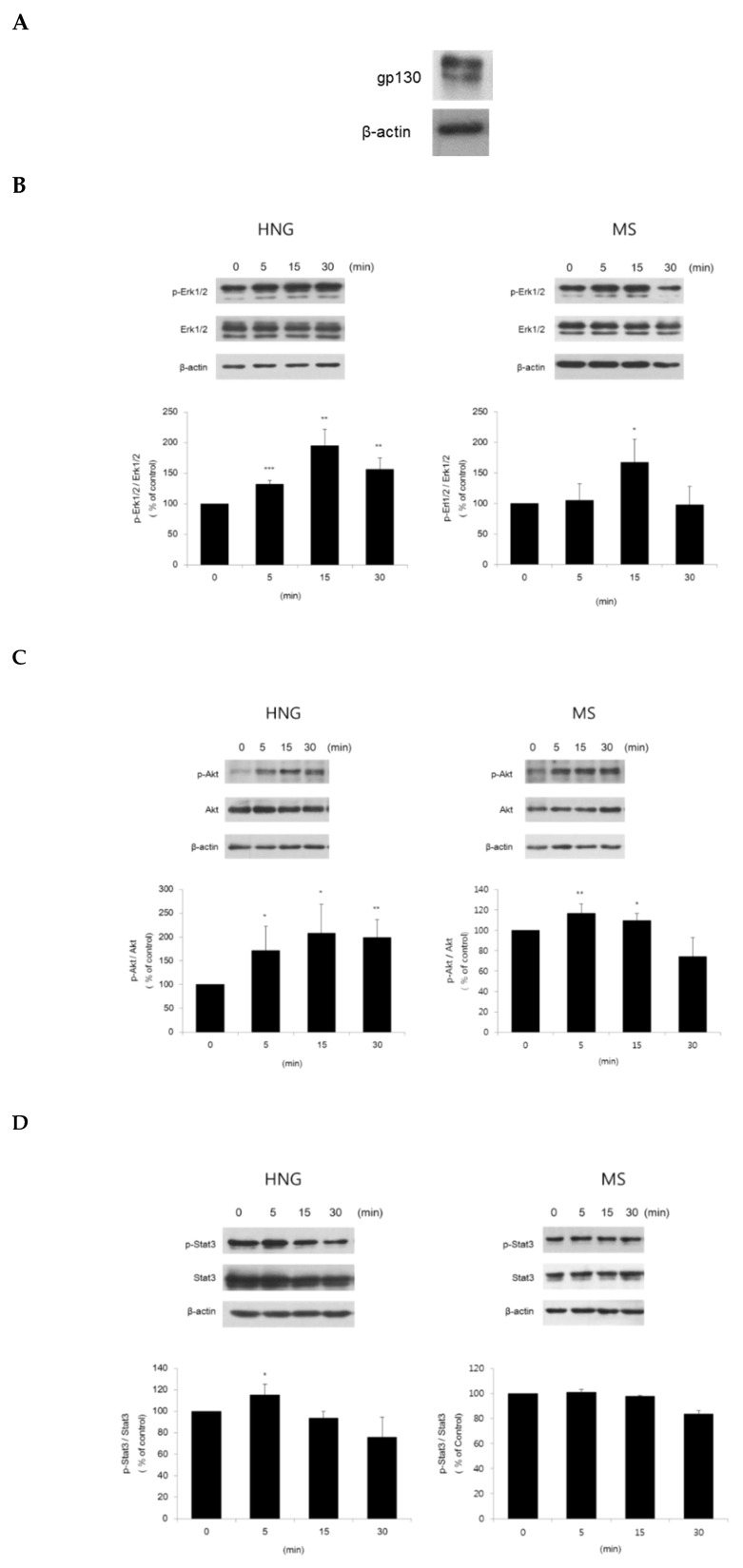
Extracellular signal-regulated kinase (Erk)1/2, Akt, and signal transducer and activator of transcription 3 (Stat3) activation through the gp130 receptor in DPCs. DPCs (1 × 10^5^ cells/mL) were seeded in Dulbecco’s modified Eagle’s medium (DMEM) supplemented with 0.5% fetal bovine serum (FBS). After 24 h, the cells were treated with HNG 200 nM for the indicated time periods. Total cell lysates were immunoblotted using anti-phospho-Erk (Thr^202^/Tyr^204^), -Akt (Ser^473^), and -Stat3 (Tyr^705^). All experiments were performed in triplicate. (**A**) Expression of the gp130 receptor, to which HNG binds. Quantification and representative Western blot of (**B**) Erk1/2 activation (**C**) Akt activation and (**D**) Stat3 activation. Data are shown as the mean ± the S.D. * *p* < 0.05, ** *p* < 0.01, *** *p* < 0.001 vs. control.

It has been reported that treatment with HNG activates Erk1/2, Akt, and Stat3 via a glycoprotein 130 (gp130) receptor in SH-SY5Y cells [21]. Minoxidil has been shown to induce proliferation by activating ERK1/2 and Akt [22]. We observed the expression of a gp130 receptor in DPCs (Figure 4A). We examined whether HNG can also activate Erk1/2, Akt, and Stat3 in DPCs. Treatment with HNG (200 nM) activated Erk1/2, Akt, and Stat3 within 5 min, respectively (Figure 4B–D). Treatment with MS (1 μM) as a positive control also activated Erk1/2 and Akt, but not Stat3 (Figure 4B–D).

### 2.5. HNG up-regulates the Expression Level of VEGF mRNA in DPCs

**Figure 5:**
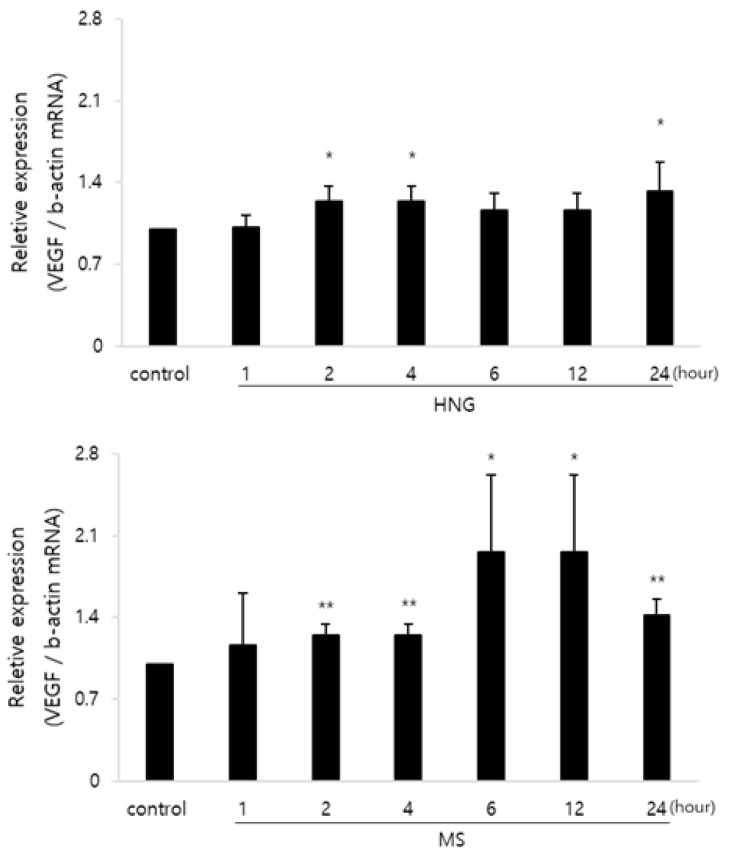
The expression level of vascular endothelial growth factor (VEGF) in DPCs treated with HNG. DPCs (1 × 10^5^ cells/mL) were seeded in DMEM supplemented with 0.5% FBS. After 24 h, the cells were treated with HNG 200 nM for the indicated time. The expression level of VEGF mRNA was evaluated using real-time PCR and normalized to β-actin mRNA. Data are shown as the mean ± S.D * *p* < 0.05, ** *p* < 0.01 vs. control.

It has been reported that VEGF is an anagen prolongation factor and a Stat3 target gene expressed in DPCs [2,7,8,28,29]. Since HNG could activate Stat3 in DPCs, we further investigated whether the expression of VEGF, one of the target genes, is up-regulated in DPCs. After 2 h of HNG treatment, the expression level of VEGF mRNA was up-regulated in DPCs where the up-regulation of the expression level of VEGF mRNA was maintained for 24 h (Figure 5). The peak up-regulation expression level of VEGF mRNA was 1.3-fold relative to control in DPC treated with HNG (Figure 5). The expression level of VEGF mRNA was also up-regulated in DPCs treated with MS used as a positive control and it was also maintained for 24 h (Figure 5).

## 3. Discussion

Here, we demonstrated that HNG promoted hair growth by facilitating both the elongation of hair-fiber length and anagen duration by preventing apoptosis. Moreover, we observed that HNG could induce dermal papilla cell proliferation by up-regulating VEGF by activating Erk1/2, Akt, and Stat3 signaling.

The hair cycle, including the anagen (growth), catagen (regression), and telogen (resting) phases, is regulated by various signaling molecules and interactions between follicular cells. Among the follicular cells, DPCs, mesenchymal derived fibroblasts, act as leading regulators in the hair cycle [11]. During anagen of hair cycle, the dermal papilla is recovered with the same number of cells in previous anagen by cell division within the dermal papilla itself and by influx of dermal sheath cells to dermal papilla [4]. Further studies are needed to determine whether HNG treatment increases emigration of the cells into dermal papilla and the level of inductivity markers such as ALP and versican [30]. Since the number of DPCs is decreased in most alopecia cases, it is meaningful for alopecia therapy that HNG increased DPC proliferation (Figure 1). In particular, anagen phase duration is accompanied by increased DPC proliferation [12]. HNG also increased the hair shaft length in the hair follicles in ex vivo experiments (Figure 2). The increase in the hair shaft length might be due to longer anagen phase duration due to the inhibition of transition from anagen to catagen phase because vibrissa follicles on anagen phase were isolated and cultured in ex vivo experiments. In an in vivo experiment, HNG could not accelerate anagen initiation, while anagen duration increased (Figure 3A,B). On the 21st day, the number of apoptotic cells on the hair follicles of HNG-treated mouse back skin was significantly less than the vehicle (Figure 3C–E). If the hair follicle transits from anagen to catagen phase, the apoptotic cells are found in the hair follicle [25,27]. As TUNEL+ cell was not found on four of ten follicles of HNG 100 nM treatment group, anagen and catagen hair follicles were estimated at 40% and 60% from Figure 3D. Figure 3D represents that HNG increases anagen duration by inhibiting transition from the anagen to catagen phase. Importantly, the quantitative histomorphometry data also demonstrates that HNG prolongs the duration of anagen (Figure 3F). The effective concentration of HNG in ex vivo assay is lower than other assays including in vitro and in vivo. According to previous our research [31,32], a possible explanation for this was as follows: in ex vivo experiments using the culture of vibrissa follicles, the culture medium containing HNG was changed every 3 days, and this practice was continued for 21 days. Therefore, the effective concentration was lower than expected, because HNG and its metabolites were concentrated in hair follicles. In our previous studies, ex vivo experiments showed efficacy at lower concentration compared to other in vitro experiments [31,32].

HNG has neuroprotective effects by activating Erk1/2, Akt, and Stat3 signaling via a gp130 receptor in SH-SY5Y neuroblastoma cells [21,33]. Here, we confirmed the expression of the gp130 receptor in DPCs (Figure 4A). The gp130 receptor is expressed and distributed in follicular keratinocytes, including matrix cells [34]. We investigated whether HNG could activate Erk1/2, Akt, and Stat3 signaling in DPCs. HNG treatment activated Erk1/2, Akt, and Stat3 in DPCs (Figure 4). In the study, we have not yet determined whether HNG activates Erk1/2, Akt and Stat3 via the gp130 receptor using siRNA or blocker of the gp130 receptor. Nonetheless, HNG seems to activate Erk1/2, Akt, and Stat3 via the gp130 known as a specific receptor for HNG as reported [21,33,35,36]. Erk1/2 and Akt are known to be associated with cell proliferation and apoptosis prevention [37,38,39,40,41,42]. The activation of Erk1/2 and Akt by HNG might increase DPC proliferation and inhibit apoptosis in the hair follicles of HNG-treated mouse back skin. Norgalanthamine and minoxidil significantly increased DPC proliferation by activating Erk1/2 and Akt [22,43]. Sinapic acid also showed hair growth-promoting effects on human hair follicle DPCs by activating AKT [44]. In addition, VEGF and hepatic growth factor (HGF) are expressed in DPCs and inhibit transition from the anagen to catagen phase, and they are Stat3 target genes [8,45,46,47]. HNG treatment increased VEGF mRNA levels in DPCs (Figure 5). Since HNG increased the phosphorylation of Stat3, DPCs might up-regulate VEGF and HGF, which seems to prolong growth phase duration. VEGF up-regulation by HNG would also increase viability and proliferation of DPCs because VEGF induces DPC proliferation [48]. VEGF is known to increase cell proliferation of outer root sheath (ORS) and DPCs via VEGF-R2 [31,47]. HNG also stimulates many cell types in the hair follicle by increasing VEGF.

In conclusion, our study provides scientific evidence regarding the applicability of HNG for the treatment of alopecia. Further studies will be needed to test its potential for clinical benefit.

## 4. Materials and Methods

### 4.1. Reagents and Antibodies

HNG, a potent analogue of humanin substituted with a glycine at the 14th of 24 amino acids, was purchased from the Peptide Institute, Inc. (Ibaraki-Shi, Osaka, Japan). HNG was dissolved in triple distilled water at a concentration of 0.1 mM, aliquoted and stored at −20 °C until use. MS, the active metabolite of minoxidil [49,50], was used as a positive control and was purchased from Sigma-Aldrich (St. Louis, MO, USA). MINOXYL™ (minoxidil 5%) was used in an in vivo experiment as a positive control, and it was purchased from the Hyundai Pharm. Co. (Gangnam-gu, Seoul, Korea). The following are the primary antibodies: anti-phospho-p44/42 MAPK antibody, anti-MAPK antibody, anti-phospho-Akt antibody, anti-Akt antibody, anti-phospho-Stat3 (Tyr^705^) antibody, and anti-Stat3 antibody were purchased from the Cell Signaling Technology (Danvers, MA, USA); mouse anti-beta actin was purchased from Sigma-Aldrich (St. Louis, MO, USA); mouse anti-GP130 antibody was purchased from Santa Cruz Biotechnology (Dallas, Texas, USA).

### 4.2. Cell Culture and Treatment

Rat vibrissa immortalized dermal papilla cell line [51] was donated by the Skin Research Institute, Amore Pacific Corporation R&D Center, South Korea. Rat vibrissa immortalized dermal papilla cells were 2D-cultured in high glucose Dulbecco’s modified Eagle’s medium (DMEM; GE Healthcare Life Science, Logan, UT, USA) supplemented with 10% fetal bovine serum (FBS; Life Technologies, Grand Island, NY, USA) and 1% antibiotic-antimycotic (Anti-Anti; Life Technologies, Carlsbad, CA, USA). Immortalized DPCs were seeded in DMEM medium containing 0.5% FBS and 1% Anti-Anti for MTT, Western blot assay, and Real-Time PCR.

### 4.3. Cell Proliferation Assay

An amount of 200 μL of immortalized DPCs (0.5 × 10^4^ cell/mL) was seeded in 96-well plates. After 24 h, DPCs were treated with humanin or MS for 72 h. Next, 50 μL of MTT solution (2 mg/mL) was added into each well. After 4 h, the supernatant from each well was discarded and 200 μL of DMSO was added. The plates were shaken to solubilize formazan crystals for 20 min. Subsequently, the optical density was measured at 540 nm by the Versamax microplate reader (Molecular Devices, Sunnyvale, CA, USA).

### 4.4. Western Blot Analysis

Immortalized DPCs were lysed with protein extraction solution (iNtRON Biotechnology, Seongnam, Gyeonggi-do, Korea) for 30 min. The supernatant was collected by centrifuging at 15,000× *g* for 15 min at 4 °C. Protein content in the cellular lysates was quantified using the Protein assay dye reagent (Bio-rad, Hercules, CA, USA). Equal lysate amounts were separated using 4–20% sodium dodecyl sulfate-polyacrylamide gel electrophoresis and transferred onto polyvinylidenefluoride membranes (GE healthcare, Little Chalfont, Buckinghamshire, UK). After blocking with 5% skimmed milk, membranes were incubated with primary antibody at 4 °C overnight. After washing three times with Tris-buffered saline containing 0.1% Tween-20, membranes were incubated with the appropriate HRP-conjugated secondary antibody at room temperature. Enhanced chemiluminescence was used to detect specific proteins. ImageJ software was used to quantify specific proteins.

### 4.5. Real-Time Quantitative Polymerase Chain Reaction (Real-Time PCR)

Total RNA was extracted with TRIzol™ Reagent (Thermo Fisher Scientific, Carlsbad, CA, USA). RNA yield and purity were determined at 260/280 nm using a Jenway 7315 spectrophotometer (Bibby Scientific, Stone, Staffordshire, UK). An equivalent amount of RNA (2 μg per sample) was used to synthesize cDNA using the MG cDNA synthesis kit (MGmed, Geumcheom-gu, Seoul, Korea). Reverse transcription was performed using random octamer at 65 °C for 5 min and MMLV reverse transcriptase at 42 °C for 30 min. The VEGF-specific primers and β-actin-specific primers were used as reference genes in the experiment, and the results are shown in Table 1. Real-time PCR was performed using iQ™SYBR^®^Green Supermix (Bio-rad, Hercules, CA, USA) in accordance with the thermal cycling protocol of iQ™SYBR^®^Green Supermix. The thermal cycling protocol conditions included the initial polymerase activation and DNA denaturation step at 95 °C for 3 min followed by the amplification step of 40 cycles consisting of denaturation at 95 °C for 10 s and annealing and extension at 55 °C for 30 s. Melt curve analysis was performed at the end of the amplification step to assess the specificity of the products formed. The relative expression level of VEGF mRNA was normalized to β-actin mRNA and calculated using the 2^−ΔΔCT^ method. The results were presented as the relative expression compared to control at the same time.

### 4.6. Animals

All animals were cared for using protocols (20180030) approved on July 17, 2018 by the Institutional Animal Care and Use Committee (IACUC) of Jeju National University. Female 6-week-old C57BL/6 mice and male 3-week-old Wistar rats were purchased from Orient Bio (Seongnam, Gyeonggi, Korea) and provided with a standard laboratory diet and water ad libitum.

### 4.7. Isolation and Culture of Rat Vibrissa Follicles

Rat vibrissa follicles in anagen phase were isolated and cultured using the method described previously [52]. Mystacial pads of Wistar rats (17 day-old) were obtained and placed in a 1:1 (*v*/*v*) solution of Earle’s balanced salts solution and phosphate-buffered saline (PBS) supplemented with 100 units of penicillin per mL and 100 mg streptomycin per mL (E/P buffer). Each vibrissa follicle was isolated by microdissection and transferred to a petri dish containing E/P buffer. Each vibrissa follicle was divided into six groups (*n* = 7) considering the size of obtained follicles between groups and cultured in each well of 24-well plates in 500 μL of Williams E medium supplemented with 2 mM l-glutamine, 10 μg insulin per mL, 10 ng hydrocortisone per mL, 100 unit penicillin per mL, and 100 μg streptomycin per mL at 37 °C in the atmosphere of 5% CO_2_/95% air. The vibrissa follicles were treated with HNG (50, 100 and 200 nM) or MS 1 µM. Thedia was refreshed every three days with HNG or MS. Photographs of the cultured rat vibrissae follicles were acquired using a stereomicroscope. The lengths of the hair follicles were measured using DP controller software (Olympus, Tokyo, Japan).

### 4.8. Hair-Growth In Vivo Experiment

It has been reported that 7-week-old mice are in telogen phase as time-scale for the murine hair cycle, and anagen phase from telogen phase is induced by hair depilation [25]. Therefore, 7-week-old female C57BL/6 mice were purchased. At the start of the mouse experiment, the mice were divided into five randomized groups (*n* = 6) and shaved to induce anagen phase. Topical treatments with HNG (50, 100, and 200 nM) or MINOXYL™ (5% minoxidil) were applied once a day for 35 days. The back skin of the mice was observed and photographed at 1, 7, 14, 21, 28, and 35 days after depilation. Quantitative results were obtained using dot matrix planimetry [53].

### 4.9. TUNEL (Tdt-Mediated dUTP-Dig Nick and Labeling) Assay and Hematoxylin and Eosin Staining

Dorsal skin of mice treated with HNG 100 nM or MINOXYL™ was dissected, fixed in 4% paraformaldehyde (Biosesang, Seongnam, Gyeonggi-do, Korea), embedded in paraffin and cut into 5 μm-thick sections for TUNEL (Tdt-mediated dUTP-Dig nick and labeling) assay and hematoxylin and eosin (H&E) staining. DeadEND™ Colorimetric TUNEL System kit (G7130 and G7360) was purchased from Promega (Madison, WI, USA) to evaluate apoptotic cells in the hair follicle. After deparaffinization, tissue sections were treated with 20 μg/mL Proteinase K solution for 30 min. Tissue sections were rinsed with distilled water (DW) and immersed in 0.3% hydrogen peroxide in methanol to block the endogenous peroxidases for 20 min. After washing with DW and PBS, tissue sections were equilibrated with equilibration buffer for 10 min. Tissue sections were incubated in a rTdT reaction mix for 60 min inside a humidified chamber. The reaction was terminated in 2× SSC for 15 min. After washing with PBS, tissue sections were incubated with the Streptavidin HRP solution for 30 min. Color was developed using the diaminobenzidine (DAB) solution, and tissue sections were counterstained with hematoxylin. For H&E staining, the tissue sections were deparaffinized and hydrated. The sections were incubated with hematoxylin for 1 min and washed with tap water for 5 min. The sections were stained with eosin for 1 min and dehydrated in xylene. Sections were mounted with Permount (Fisher Scientific, Hampton, NH, USA).

### 4.10. Statistical Analysis

Data are shown as mean ± standard deviation (S.D) or mean ± standard error (S.E) of at least triplicate experiments. The significant differences were determined by Student’s *t*-test, using the SigmaStat software ver. 3.5 (San Jose, CA, USA). A *p*-value < 0.05 was considered statistically significant.

## Figures and Tables

**Table 1:** Primers for rat β-actin and vascular endothelial growth factor (VEGF).

Primer Name	Sequence (from *5′* to *3′*)
VEGF *forward*	*5′-AAC GAA AGC GCA AGA AAT CC-3′*
VEGF *reverse*	*5′-GCT CAC AGT GAA CGC TCC AG-3′*
β-actin *forward*	*5′-TCC TGG CCT CAC TGT CCA C-3′*
β-actin *reverse*	*5′-GGG CCG GAC TCA TCG TAC T-3′*
